# Molecular Characterization of *Anaplasma* spp. among Dairy, Cashmere, and Meat Goats in Shaanxi Province, Northwestern China

**DOI:** 10.3390/ani12121566

**Published:** 2022-06-17

**Authors:** Xin Yang, Mingzhe Fu, Zhengqing Yu, Junwei Wang, Junke Song, Guanghui Zhao

**Affiliations:** 1 College of Veterinary Medicine, Northwest A&F University, Yangling, Xianyang 712100, China; xinyang@nwafu.edu.cn (X.Y.); fmzhe@163.com (M.F.); w13186039897@163.com (J.W.); 2 College of Veterinary Medicine, Nanjing Agricultural University, Nanjing 210095, China; babyyuzh@126.com

**Keywords:** anaplasmosis, 16S rRNA gene, *msp4* gene, prevalence, blood DNA

## Abstract

**Simple Summary:**

Anaplasmosis is an important tick-borne disease caused by *Anaplasma* spp., significantly threating public health safety and breeding industry. The present study reported the occurrence of *Anaplasma* infection among dairy, cashmere, and meat goats in Shaanxi province, northwestern China, with the total prevalence of 58.5% (298/509) in goats. *Anaplasma phagocytophilum*, *A. bovis*, and *A. ovis* were the dominant species in meat, dairy, and cashmere goats, respectively, with the absence of *A. ovis* in meat goats. Furthermore, the different influencing factors (production categories, species, regions, and ages) were analyzed, and statistically significant differences were found. Frequent occurrence of *Anaplasma* in this study indicated One Health-based intervention approaches were urgently needed to block the transmission between humans and animals.

**Abstract:**

*Anaplasma* spp. are important tick-borne pathogens endangering the health of humans and various animals. Although several studies have reported *Anaplasma* infection in livestock in China, little is known about the impact of production categories on the occurrence of *Anaplasma* species. In the present study, PCR tools targeting the 16S rRNA and *msp4* genes were applied to investigate the prevalence of *Anaplasma* spp. in 509 blood samples of dairy (*n* = 249), cashmere (*n* = 139), and meat (*n* = 121) goats from Shaanxi province. The prevalence of *Anaplasma* spp. was 58.5% (298/509) in goats, and significant differences (*p* < 0.001) were identified in the prevalence among production categories, with the highest in meat goats (84.3%, 102/121), followed by cashmere goats (58.3%, 81/139) and dairy goats (46.2%, 115/249). Significant differences (*p* < 0.001) in prevalence were also found among sampling sites and age groups. Meanwhile, the prevalence was 36.9% (188/509) for *A. phagocytophilum*, 36.1% (184/509) for *A. bovis*, and 11.0% (56/509) for *A. ovis*, and significant differences (*p* < 0.001) in prevalence of *A. phagocytophilum*, *A. bovis* and *A. ovis* were recognized among production categories and sampling sites. *A. phagocytophilum*, *A. bovis* and *A. ovis* were dominant species in meat, dairy, and cashmere goats, respectively, and *A. ovis* was absent in meat goats. Co-infections were found in 124 (24.4%) investigated samples. Goats aged < 2, 3–6, and 7–12 months, and goats from Qingjian and Zhenba were risk factors associated with the occurrence of *Anaplasma*. Phylogenetic analysis indicated separate clades for the distribution of *A. phagocytophilum* from different ruminant, reflecting potential host adaption within this species. This study reported the colonization occurrence of *Anaplasma* spp. among production categories in goats in Shaanxi province and enriched our knowledge on the transmission of *Anaplasma* spp. in goats in China. Considering the existence of zoonotic *A. phagocytophilum* in goats in this study and previous reports, interventions based on One Health are needed to be developed to control the transmission of *Anaplasma* spp. between humans and animals.

## 1. Introduction

*Anaplasma* spp. are emerging tick-borne pathogens infecting humans and animals which largely distribute in tropical, subtropical, and some temperate regions [1,2,3]. Currently, *Anaplasma* *phagocytophilum*, *A. bovis*, *A. ovis*, *A. marginale*, *A. centrale*, *A. platys*, and *A. capra* are recognized species within *Anaplasma* [1,4]. Of them, *A*. *phagocytophilum*, *A. bovis*, and *A. ovis* are common pathogenic species causing anaplasmosis. *A. phagocytophilum* can infect neutrophils of humans and various animals [1,5], causing human granulocytic anaplasmosis (HGA) and tick-borne fever in ruminants [6,7]. *A. bovis* is mainly found in mononuclear cells of many animals, resulting in anaplasmosis with fever, weight loss, depression, abortion, or even death [8]. *A. ovis* can infect erythrocytes of small ruminant [2,9], leading to symptoms including hemolytic anemia and fever [10].

As important tick-borne pathogens, *Anaplasma* spp. significantly affect the health of humans and animals [9]. *A. phagocytophilum* majorly leads to HGA, a common disease leading to fever in patients. HGA was first reported in an American patient with tick bite history in 1990, and subsequently found in over 14 European countries [6]. According to the data from CDC of USA and Morbidity and Mortality Weekly Reports, over 15,952 HGA cases have been reported in America since 1995. The occurrence of HGA cases increased 12 times from 2001 to 2011, and the disease caused life threatening illness and death in approximately 3% and 1% of infection cases, respectively [11,12,13]. Meanwhile, the World Organization for Animal Health (WOAH) has listed anaplasmosis as a notifiable disease due to its socio-economic impact and international trade restrictions [14].

Knowledge on the distribution and composition of *Anaplasma* spp. can shed the light for the prevention and control of anaplasmosis. With the application of PCR-based techniques targeting the 16S rRNA, *msp4*, *gltA*, and *groEL* genes, single and/or mixed infections of *Anaplasma* species, such as *A. phagocytophilum*, *A. bovis*, and *A. ovis*, have been recognized in various animals, ticks, and humans [3,15,16,17].

Recently, several studies have reported *Anaplasma* infection in livestock in China [18,19,20,21,22,23,24], however, little is known on the occurrence of *Anaplasma* spp. among production categories. Goats are important economic livestock in China, providing meat, cashmere, and dairy with high quality in daily life. Shaanxi province is one of the main goat breeding areas in China, with a total of 7.22 million goats in 2020 (http://data.stats.gov.cn/, accessed on 20 May 2022). Notably, there exist three main production categories (dairy, cashmere, and meat) for goats in Shaanxi, and the dairy goat stocks and goat milk production of Shaanxi province account for 48.2% and 61.4% of the national total, respectively (http://nynct.shaanxi.gov.cn/, accessed on 20 May 2022). To understand the distribution characteristics of *Anaplasma* spp. among production categories in goats in Shaanxi province, the present study investigated the prevalence of *A. phagocytophilum*, *A. bovis*, and *A. ovis* in dairy, cashmere, and meat goats of Shaanxi province in northwestern China, and assessed the zoonotic potential of these pathogens in goats.

## 2. Materials and Methods

### 2.1. Sampling

**Figure 1:**
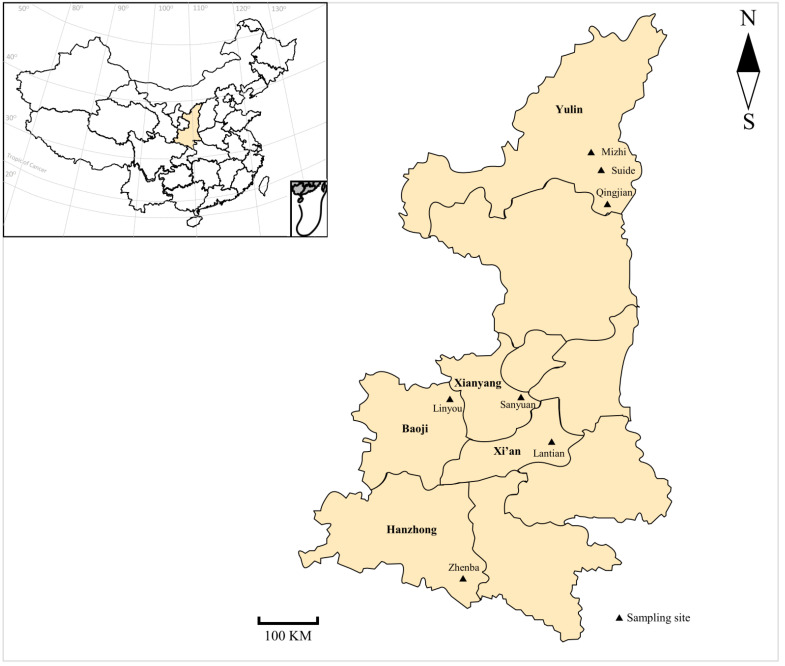
Geographical distribution of sampling sites in Shaanxi province in the present study.

To investigate the prevalence and species composition of *Anaplasma* in meat, cashmere, and dairy goats in Shaanxi province, we collected samples from representative farms in seven main breeding sampling sites, with 10~20% of animals sampled for each farm (Figure 1). From April to July 2017, a total of 509 blood samples of dairy (*n* = 249), cashmere (*n* = 139), and meat (*n* = 121) goats were collected from 15 representative farms (Table 1). Due to the larger scale of dairy goats compared with other two production categories, we collected more farms and more samples from dairy goats (Table 1). Blood samples were collected from the jugular vein of each animal and placed into separate tubes containing 1.5% Ethylenediaminetetraacetic acid tripotassium (EDTA-3K) with basic information (e.g., sampling sites, breeds, and ages), immediately transported to the department of parasitology of Northwest A&F University under cool condition, and then kept at 4 °C for further analysis.

### 2.2. Genomic DNA Extraction

The Blood Genomic DNA Isolation Kit (Sangon Biotech, Shanghai, China) was applied to extract genomic DNA (gDNA) samples from approximately 100 μL blood of each sample according to the procedures of the manufacturer, and the gDNA samples were kept at −20 °C until further analysis. Meanwhile, *Anaplasma* negative blood samples preserved in our lab were also applied for genomic DNA extraction to be used as negative control in PCR amplification.

### 2.3. PCR Amplification

The occurrence of *A. phagocytophilum* and *A. bovis* was identified using nested-PCR targeting a ~551 bp fragment and a ~641 bp fragment of the 16S rRNA gene as reported, respectively [25,26] (Table 2). Nested PCRs were carried out in a 25 μL reaction mixture containing 1 × *Ex Taq* Buffer (Mg^2+^ free), 2 mM MgCl_2_, 0.2 mM dNTP Mixture, 1 U TaKaRa *Ex Taq*, 0.4 μΜ each primer, 1 μL gDNA for the primary PCR, or 1 μL primary PCR product for the secondary PCR under the following conditions for both primary and secondary PCRs: an initial denaturing at 94 °C for 5 min, followed by 35 cycles of 94 °C for 30 s, 55 °C for 1 min, and 72 °C for 1 min, and a final extension at 72 °C for 10 min. *A. ovis* DNA was identified by PCR amplification targeting a ~867 bp fragment of the *msp4* gene [27] (Table 2). PCRs were conducted in a 25 μL reaction mixture containing 1 × *Ex Taq* Buffer (Mg^2+^ free), 2 mM MgCl_2_, 0.2 mM dNTP Mixture, 1 U TaKaRa *Ex Taq*, 0.4 μΜ each primer, and 1 μL gDNA under the following conditions: an initial denaturing at 94 °C for 30 s, followed by 40 cycles of 94 °C for 30 s, 60 °C for 30 s, and 68 °C for 1 min, and a final extension at 68 °C for 10 min. A negative control without *Anaplasma* was used in each PCR amplification. Positive PCR products of the *msp4* gene and secondary nested-PCR products of the 16S rRNA gene will appear a band of the expected size under a UV transilluminator after 1% agarose gel electrophoresis. A Good Laboratory Practice was followed to avoid contamination in each step during the whole experiment [28].

### 2.4. Sequencing and Sequence Analysis

All positive amplicons were sequenced at forward direction by Sangon Biotech (Shanghai, China) using an ABI PRISM 3730XL DNA Analyzer (Applied Biosystems, Bedford, MA, USA). The obtained sequences were identified to be *A. phagocytophilum* 16S rRNA gene, *A. bovis* 16S rRNA gene, or *A. ovis msp4* gene by BLAST analysis within NCBI (https://blast.ncbi.nlm.nih.gov/Blast.cgi; accessed on 15 April 2021). To assess the phylogenetic placement of *Anaplasma* species found in this study, phylogenetic trees were constructed by using the neighbor-joining (NJ) method with the Kimura 2-parameter model and the calculation of substitution rates with the bootstrap evaluation of 1000 replicates within software MEGA V6.0 [29].

### 2.5. Statistical Analysis

Data analysis was conducted using the package R V4.0.5 (https://www.r-project.org/; accessed on 7 July 2021) and RStudio V1.1.463 (http://rstudio.com/; accessed on 7 July 2021). A chi-square test was applied to analyze differences in prevalence of *Anaplasma* spp. by production categories, age groups, or sampling sites. Logistic regression was used to analyze the association between risk factors and the occurrence of *Anaplasma* spp. in dairy, cashmere, and meat goats. Initially, univariate analysis was used to assess the strength of association. Then, a multivariate model was built using variables with *p* ≤ 0.2, with *p* < 0.05 being recognized as significant in the final model. Odds ratio (OR) with 95% confidence intervals (CI) was analyzed for the identification of risk factors for the occurrence of *Anaplasma* spp. in goats.

### 2.6. Nucleotide Sequence Accession Numbers

Representative nucleotide sequences of *A. phagocytophilum* 16S rRNA gene, *A. bovis* 16S rRNA gene, and *A. ovis msp4* gene in the present study were available in GenBank™ under the accession numbers of MZ489423-MZ489426, MZ489427-MZ489430, and MZ502497-MZ502499, respectively.

## 3. Results

### 3.1. Prevalence of Anaplasma in Goats in Shaanxi Province

Of the 509 blood samples, a total of 298 (58.5%) samples were positive for *Anaplasma* spp. in goats based on the PCR-sequencing tools targeting the 16S rRNA gene and the *msp4* gene (Table 3). Among the three production categories in goats, the highest prevalence (84.3%, 102/121) of *Anaplasma* spp. was found in meat goats, which was significantly higher than that in cashmere goats (58.3%, 81/139) (χ^2^ = 21.018, *df* = 1, *p* < 0.001) and dairy goats (46.2%, 115/249) (χ^2^ = 48.773, *df* = 1, *p* < 0.001), and significant difference was also identified between cashmere goats and dairy goats (χ^2^ = 5.215, *df* = 1, *p* = 0.022) (Table 4 and Table 5). Meanwhile, significant differences in prevalence were also identified among age groups (χ^2^ = 17.659, *df* = 3, *p* = 0.001) (Table 5), with the highest prevalence (80.0%, 12/15) in goats aged <2 months, followed by 7–12 months (75.0%, 45/60), >12 months (58.8%, 197/335), and 3–6 months (44.4%, 44/99) (Table 3).

In the univariate analysis, there existed association between dairy goat (OR = 5.26, *p* < 0.001) and occurrence of *Anaplasma*, especially for *A. phagocytophilum* (OR = 0.52, *p* = 0.001) and *A. ovis* (OR = 0.07, *p* < 0.001), and there also existed association between meat goat (OR = 0.36, *p* < 0.001) and occurrence of *Anaplasma*, especially for *A. phagocytophilum* (OR = 9.10, *p* < 0.001) and *A. bovis* (OR = 2.40, *p* < 0.001). Meanwhile, goats aged 3–6 months (OR = 0.49, *p* = 0.002) and 7–12 months (OR = 2.32, *p* = 0.007) were more likely to be infected with *Anaplasma*, especially for *A. phagocytophilum* (OR = 0.58, *p* = 0.028) and *A. bovis* (OR = 0.53, *p* = 0.013) for goats from 3–6 months, and *A. ovis* (OR = 2.95, *p* = 0.002) for goats from 7–12 months. In addition, goats from Lantian (OR = 0.35, *p* < 0.001), Qingjian (OR = 2.95, *p* = 0.008), and Zhenba (OR = 5.26, *p* < 0.001) were more likely to be infected with *Anaplasma*, especially for *A. phagocytophilum* (OR = 0.42, *p* = 0.031), *A. bovis* (OR = 2.76, *p* = 0.003), and *A. ovis* (OR = 9.74, *p* < 0.001) for goats from Qingjian, *A. phagocytophilum* (OR = 9.10, *p* < 0.001) and *A. bovis* (OR = 2.40, *p* < 0.001) for goats from Zhenba, *A. phagocytophilum* (OR = 0.64, *p* = 0.045) for goats from Lantian (Table 6). Further analysis applying the multivariate model indicated that there was no association between production categories and the occurrence of *Anaplasma*. However, goats aged < 2 months (OR = 0.52, *p* = 0.004), 3–6 months (OR = 0.26, *p* = 0.017), and 7–12 (OR = 0.27, *p* < 0.001) months were risk factors associated with the occurrence of *Anaplasma*, especially for *A. bovis* (OR = 5.06, *p* < 0.001) for goats aged < 2 months. In addition, goats from Qingjian (OR = 9.80, *p* = 0.004) and Zhenba (OR = 8.94, *p* < 0.001) were risk factors associated with the occurrence of *Anaplasma*, especially for *A. phagocytophilum* (OR = 12.84, *p* < 0.001) for goats from Zhenba (Table 6).

### 3.2. Anaplasma phagocytophilum in Goats in Shaanxi Province

In the present study, the prevalence of *A. phagocytophilum* in goats was 36.9% (188/509) (Table 3). All the production categories were positive for *A. phagocytophilum* with the highest prevalence in meat goats (75.2%, 91/121), followed by dairy goats (29.3%, 73/249) and cashmere goats (17.3%, 24/139), and significant differences were found among production categories (χ^2^ = 105.376, *df* = 2, *p* < 0.001) (Table 3 and Table 5). Significant differences in the prevalence of *A. phagocytophilum* were also found among sampling sites (χ^2^ = 187.211, *df* = 14, *p* < 0.001). Except for Farm 1, 4, 7, 8, and 9, all the remaining 10 sampling sites were positive for *A. phagocytophilum*, with the highest prevalence in Farm 14 (84.6%) and lowest in Farm 3 (5.9%), and significant differences in prevalence were identified among these sampling sites (χ^2^ = 96.340, *df* = 9, *p* < 0.001) (Table 3 and Table 5). Besides, *A. phagocytophilum* infection was frequently identified in all age groups of goats, with the highest prevalence in animals aged 7–12 months (43.3%, 26/60), followed by >12 months (39.1%, 131/335), 3–6 months (27.3%, 27/99), and <2 months (26.7%, 4/15) (Table 3).

**Figure 2:**
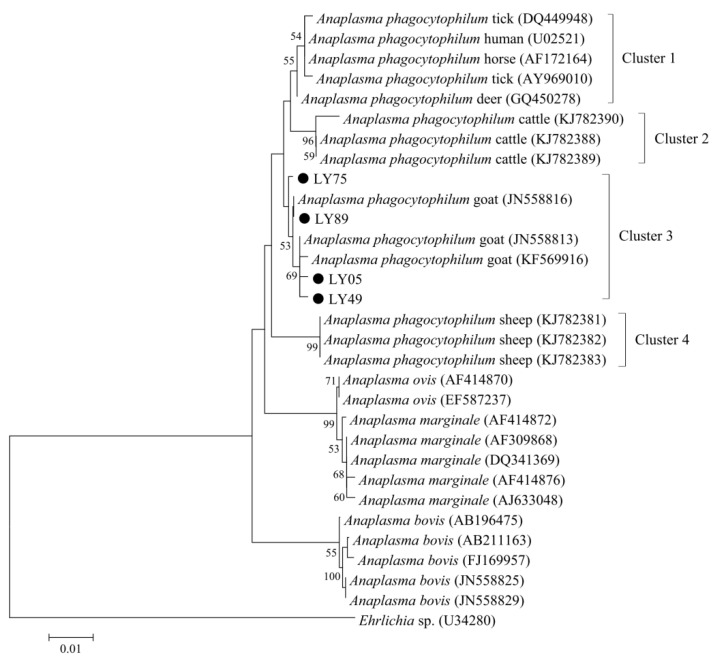
Phylogenetic relationships of *Anaplasma phagocytophilum* in the present study (black circle before the sample name) with reference sequences from GenBank^TM^ based on the sequence analysis of the 16S rRNA gene by neighbor-joining analysis using the Kimura 2-parameter model. Bootstrap values (>50) are indicated at the nodes. Scale bar indicates 0.01 nucleotide substitutions/site. *Ehrlichia* sp. (U34280) is used as the outgroup.

To assess the genetic diversity of *A. phagocytophilum* in goats, all the positive samples for *A. phagocytophilum* at the 16S rRNA locus were sequenced, and a total of 184 sequences were obtained in the present study. Based on the BLAST analysis at NCBI and sequence alignment in MEGA V6.0, four types of *A. phagocytophilum* 16S rRNA sequences, namely LY05 (6), LY49 (3), LY75 (170), and LY89 (5), were identified in goats, with 99.2–99.6% sequence identity among these sequence types. Phylogenetic analysis indicated that four *A. phagocytophilum* 16S rRNA sequences in the present study and other reference sequences from goats were included in the same clade, while sequences from cattle and sheep formed other two separated clades (Figure 2).

### 3.3. Anaplasma bovis in Goats in Shaanxi Province

In the present study, the prevalence of *A. bovis* in goats was 36.1% (184/509) (Table 3). All the production categories were positive for *A. bovis* with the highest in meat goats (52.1%, 63/121), followed by dairy goats (33.3%, 83/249) and cashmere goats (27.3%, 38/139), and significant differences were found among production categories (χ^2^ = 18.812, *df* = 2, *p* < 0.001) (Table 3 and Table 5). Significant differences in the prevalence of *A. bovis* were also found among sampling sites (χ^2^ = 126.079, *df* = 14, *p* < 0.001). Except for Farms 1 and 4, all the remaining 13 sampling sites were positive for *A. bovis*, with the highest prevalence in Farm 13 (82.5%) and lowest in Farm 8 (4.5%), and significant differences were identified among these sampling sites (χ^2^ = 98.871, *df* = 12, *p* < 0.001) (Table 3 and Table 5). Meanwhile, *A. bovis* infection was commonly found in all age groups of goats, with the highest prevalence in animals aged <2 months (80.0%, 12/15), followed by 7–12 months (45.0%, 27/60), >12 months (35.8%, 120/335), and 3–6 months (25.3%, 25/99) (Table 3).

**Figure 3:**
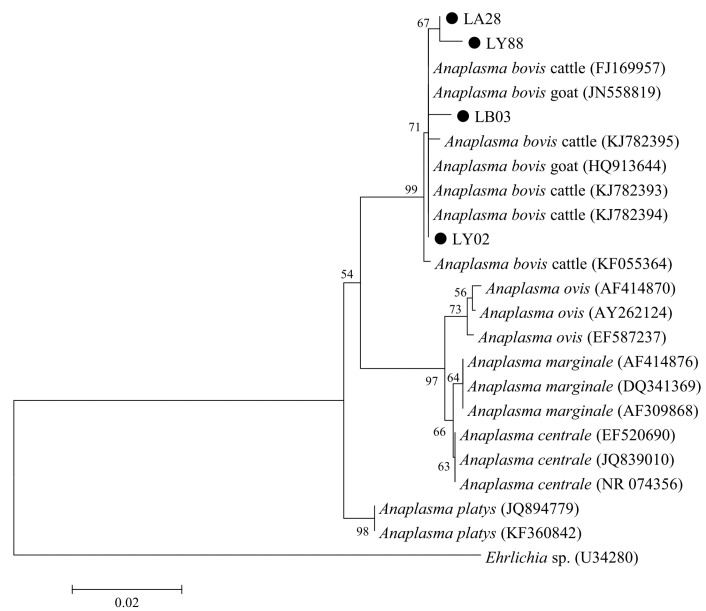
Phylogenetic relationships of *Anaplasma bovis* in the present study (black circle before the sample name) with reference sequences from GenBank™ based on the sequence analysis of the 16S rRNA gene by neighbor-joining analysis using the Kimura 2-parameter model. Bootstrap values (>50) are indicated at the nodes. Scale bar indicates 0.02 nucleotide substitutions/site. *Ehrlichia* sp. (U34280) is used as the outgroup.

Further, a total of 184 samples positive for *A. bovis* were successfully sequenced, and the obtained 184 sequences formed four sequence types, namely LA28 (4), LB03 (2), LY02 (172), and LY88 (6), with 98.7–99.7% sequence identities to each other. Phylogenetic analysis indicated that *A. bovis* samples from goats and cattle were included in the same clade (Figure 3).

### 3.4. Anaplasma ovis in Goats in Shaanxi Province

The prevalence of *A. ovis* in goats was 11% (56/509) in the present study, with the highest prevalence of 37.4% (cashmere) to the lowest of 0% (meat). Significant differences in prevalence were found among three production categories (χ^2^ = 136.408, *df* = 2, *p* < 0.001) (Table 3 and Table 5). Meanwhile, significant differences in the prevalence of *A. ovis* were also found among sampling sites (χ^2^ = 167.124, *df* = 14, *p* < 0.001). Five farms were positive for *A. ovis*, with the highest prevalence in Farm 9 (54.1%) and lowest in Farm 6 (4.4%), and significant differences were identified among these sampling sites (χ^2^ = 48.265, *df* = 4, *p* < 0.001) (Table 3 and Table 5). Meanwhile, *A. ovis* infection was frequently identified in all age groups of goats, with the highest prevalence in animals aged <2 months (26.7%, 4/15), followed by 7–12 months (23.3%, 14/60), 3–6 months (14.1%, 14/99), and >12 months (7.2%, 24/335) (Table 3).

**Figure 4:**
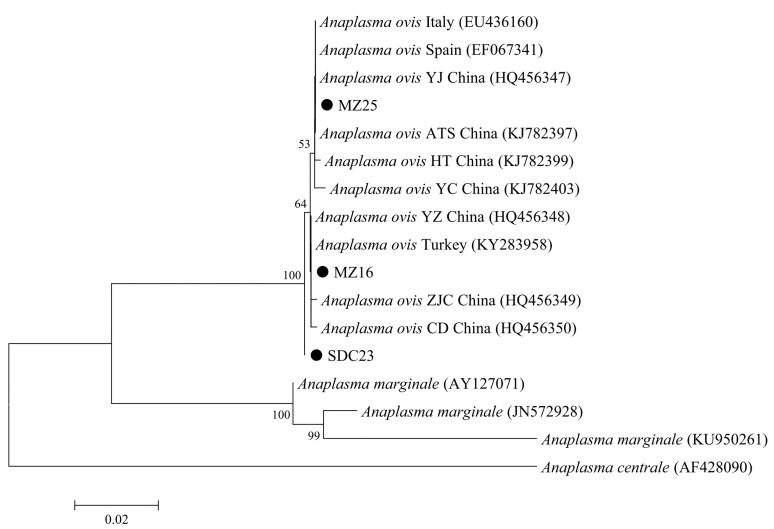
Phylogenetic relationships of *Anaplasma ovis* in the present study (black circle before the sample name) with reference sequences from GenBank^TM^ based on the sequence analysis of the *msp4* gene by neighbor-joining analysis using the Kimura 2-parameter model. Bootstrap values (>50) are indicated at the nodes. Scale bar indicates 0.02 nucleotide substitutions/site. *Anaplasma centrale* (AF428090) is used as the outgroup.

Based on the PCR-sequencing of *A. ovis msp4* gene, a total of three sequence types, namely MZ16 (30), MZ25 (18), and SDC23 (8), were recognized in the obtained 56 sequences, with 99.7–99.8% sequence similarity among these sequence types. Further phylogenetic analysis based on the *msp4* gene indicated that MZ16, MZ25, SDC23, and reference sequences were included in the same clade, showing high sequence similarity among *A. ovis* from divergent areas in China (Figure 4).

## 4. Discussion

*Anaplasma* spp. are important zoonotic pathogens with worldwide distribution in humans and various animals [4,8,19,25,30,31,32,33]. To understand the occurrence of *Anaplasma* spp. among production categories, the present study investigated the prevalence of *A. phagocytophilum*, *A. bovis*, and *A. ovis* in dairy, cashmere, and meat goats in seven sampling sites within five main breeding areas in Shaanxi, northwestern China by using PCR-sequencing tools based on the 16S rRNA gene and the *msp4* gene. Of the 509 blood samples, a total of 298 (58.5%) samples were positive for *Anaplasma* spp. in goats in the present study, and statistically significant differences were found in the prevalence of *Anaplasma* spp. among production categories.

*A. phagocytophilum* is recognized as one emerging tick-borne pathogen of public health in humans [34]. Besides humans, a variety of animals, e.g., cattle, sheep, goats, deer, horses, cats, dogs, and rats are also susceptible to this pathogen, and infected hosts can show a series of symptoms, including high fever, anorexia, and weight loss [20,24,35,36,37,38]. In the present study, the prevalence of *A. phagocytophilum* in goats was 36.9% (188/509) (Table 2), which was consistent with that in goats in Gansu (38.5%) [39], but higher than that in other provinces in China (5.7–30.8%) [18,19,20,21,24]. Recently, one study reported a higher average prevalence of 71.5% for *A. phagocytophilum* in goats in Weinan city, Shaanxi [22]. The differences in the occurrence rates of *A. phagocytophilum* in goats were likely affected by multiple factors, including different detection methods, geographic regions, sampling sizes, and animal management practice [40,41].

Previously, several reports indicated the existence of differences in host infectivity among divergent *A. phagocytophilum* strains [42,43,44]. Phylogenetic analysis indicated that four *A. phagocytophilum* 16S rRNA sequences in the present study and other reference sequences from goats were included in the same clade, while sequences from cattle and sheep formed two separated clades, suggesting the possible host adaptation for the distribution of *A. phagocytophilum* in these domesticated animals (Figure 2). Similar results have also been identified in *A. phagocytophilum*-positive samples from sheep and cattle in one previous study, reflected by the formation of separate clades for this pathogen from sheep and cattle by phylogenetic analysis [19].

In the present study, the 16S rRNA gene was applied for the genetic characterization of *A. phagocytophilum* of goats and found variants among different domesticated animals by phylogenetic analysis. The conserved 16S rRNA gene has been widely applied to detect *A. phagocytophilum* infection in humans and ruminants, identifying variants within this pathogen. However, the variants of the 16S rRNA are controversial due to the low discriminatory power. Therefore, several molecular markers, such as *groESL*, *ankA*, and *Msp2*, were tested and likely showed better function in distinguishing variant of divergent pathogenicity or origins compared with 16S rRNA [45]. The *drhm* gene was previously recognized as a maker for the pathogenicity of *A. phagocytophilum*, but it was not useful for several European *A. phagocytophilum* strains [46]. To increase the resolution in the characterization of *A. phagocytophilum*, variable number tandem repeat (VNTR), multi-locus sequence typing (MLST), and other multi-locus sequence analysis were used, which could significantly contribute to the comprehensive understanding of *A. phagocytophilum* [45,47].

*A. bovis* can infect a variety of mammals, including cattle, sheep, goats, deer, cats, and dogs [18,25,48,49,50,51]. In the present study, the prevalence of *A. bovis* in goats was 36.1% (184/509) (Table 2), which was lower than that in Tunisia (72.0%) [8], but higher than that in other regions in China (12.3–20.3%) [18,21]. Previously, some studies reported lower prevalence of 9.5% for *A. bovis* in goats in Xi’an city, Shaanxi [52], and higher prevalence of 62.1% in Weinan city, Shaanxi [22]. Animal management practices with the good application of drugs and procedures against ectoparasite possibly contribute to the low prevalence of *A. bovis* in goats since the common occurrence of this pathogen in several ticks, including *Rh. microplus*, *Ha. longicornis*, and *Ixode crenulatus* [41,53]. Contrary to the distribution of *A. phagocytophilum*, *A. bovis* samples from goats and cattle were included in the same clade by phylogenetic analysis, suggesting the possible absence of host segregation for the distribution of *A. bovis* (Figure 3), which was in accordance with the previous report in sheep and cattle [19]. Nevertheless, potential geographic differences for the distribution of *A. bovis* were identified in goats in China [22].

The average prevalence of *A. ovis* (11%, 56/509) in goats in the present study was lower than that in France (52%) [54], Mongolia (71.3%) [55], and other regions in China, e.g., Guizhou (17.8%), Xinjiang (40.5%), Zhejiang (26.3%) [18,19], but higher than that in Heilongjiang (2.6%), Henan (8.7%), and Hubei (7.2%) in China [18,21]. One previous study reported lower prevalence of *A. ovis* (0.9%) in goats in Xi’an city, Shaanxi [56], and the other study found higher prevalence of *A. ovis* (25.2%) in goats in Weinan city, Shaanxi [22]. Sampling sites, breeds, animal management practices, as well as other multiple factors, may lead to the differences in the prevalence of *A. ovis* among studies [40,41]. Notably, the occurrence of ticks (e.g., *Rhipicephalus bursat*) may also influence the prevalence of *A. ovis* in goats [54]. Among those three production categories in the present study, *A. ovis* was absent in meat goats, which was likely caused by the specific species composition of ticks in the studied regions due to the possible biased distribution of *Anaplasma* spp. among divergent ticks [9,54]. As an important major surface protein of *Anaplasma* spp., the *msp4* gene is likely to have higher evolution rate under the selective pressure of host immune system compared with other genes [27]. In the present study, limited variation was observed within the sequences of the *msp4* gene among *A. ovis* isolates, which was in accordance with the previous reports, reflected by low genetic diversity of *A. ovis* isolates from Xinjiang and Shaanxi [19,22].

Notably, co-infections of two to three *Anaplasma* species were identified in the present study (24.4%, 124/509), which was also commonly found in previous reports [18,22,49]. Significant differences were found among co-infections of *A. phagocytophilum* and *A. bovis*, *A. bovis* and *A. ovis*, and *A. phagocytophilum* and *A. ovis* (χ^2^ = 134.594, *df* = 2, *p* < 0.001), with the co-infection of *A. phagocytophilum* and *A. bovis* being the dominant one (83.1%, 103/124). Interesting, co-infection of *A. phagocytophilum*, *A. bovis*, and *A. ovis* was recognized in 6 samples from dairy and cashmere goats, which has also been reported in 6 samples from goats in a previous report [18].

Previous reports indicated that the age was an influence factor for the infection of *Anaplasma* [8,23]. In the present study, significant difference was found among ages, with the highest prevalence in young animals under 2 months, which may be caused by low immunity or few samples tested in these animals. However, a higher prevalence of *Anaplasma* species in old goats (≥2 years) than young goats (<2 years) was found in one study in Anhui, China [23]. The difference in prevalence could be old animals being exposed to tick infection for a longer time. More investigations with a wide age range and geographic region settings are needed to further understand the influence of ages on *Anaplasma* infection.

## 5. Conclusions

In the present study, *Anaplasma* spp. were found in goats, with a prevalence of 58.5%, and significant differences in prevalence were found in animals from different production categories and age groups. *A. phagocytophilum*, *A. bovis*, and *A. ovis* were dominant species in meat, dairy, and cashmere goats, respectively, and *A. ovis* was absent in meat goats. Meanwhile, co-infections with two or three pathogens were frequently recognized. Frequent occurrence of *A. phagocytophilum*, *A. bovis*, and *A. ovis* in goats in this study indicates that there possibly exist potential risks for the zoonotic transmission between humans and animals due to the appearance of those pathogens in humans in previous reports. These findings can provide baseline information for the understanding of the transmission and zoonotic potential of *Anaplasma* spp. in goats. Considering the public health risks to humans of zoonotic *A. phagocytophilum* being found in goats, interventions based on One Health, such as eliminating ticks, maintaining good farm husbandry practices, and keeping personal hygiene, are needed to reduce the transmission of this pathogen from goats to humans and other animals. Certainly, there exist several limitations in the present work, and several questions should be addressed in future studies. For example, did the distribution and species composition of ticks effect the prevalence of *Anaplasma* spp. in goats? Are tick species and/or burdens related to the distribution of *Anaplasma* species or not? Why were young children more susceptible to *Anaplasma* then elder animals? Answers to these questions will assist us to comprehensively illustrate the transmission of *Anaplasma* between humans and animals.

## Figures and Tables

**Table 1:** Information on the samples collected in the present study.

Categories	Sampling Sites	Farms	Management	No. Total	No. Tested
Guanzhong dairy goat	Sanyuan	Farm 1	Good	200	26
	Lantian	Farm 2	Medium	280	39
		Farm 3	Good	150	17
		Farm 4	Good	146	15
		Farm 5	Poor	240	27
	Linyou	Farm 6	Poor	500	91
		Farm 7	Good	100	12
Saanen dairy goat	Sanyuan	Farm 8	Good	120	22
Shaanbei white cashmere goat	Mizhi	Farm 9	Medium	200	37
	Suide	Farm 10	Poor	220	39
		Farm 11	Good	200	24
	Qingjian	Farm 12	Medium	200	39
Shaannan white goat	Zhenba	Farm 13	Poor	230	40
		Farm 14	Poor	210	39
		Farm 15	Poor	210	42

Good: good animal husbandry practice with regular usage of drugs against ectoparasite; Medium: medium animal husbandry practice with random usage of drugs against ectoparasite; Poor: poor animal husbandry practice with little usage of drugs against ectoparasite.

**Table 2:** Primers for PCR analysis in the present study.

Pathogen	Target Gene	Primer	Sequence (5′–3′)	Amplicon Size (bp)	Reference
*A. phagocytophilum*	16S rRNA	EE1	TCCTGGCTCAGAACGAACGCTGGCGGC	1430	[25,26]
		EE2	AGTCACTGACCCAACCTTAAATGGCTG		
		SSAP2f	GCTGAATGTGGGGATAATTTAT	641	
		SSAP2r	ATGGCTGCTTCCTTTCGGTTA		
*A. bovis*	16S rRNA	EE1	TCCTGGCTCAGAACGAACGCTGGCGGC	1430	[25,26]
		EE2	AGTCACTGACCCAACCTTAAATGGCTG		
		AB1f	CTCGTAGCTTGCTATGAGAAC	551	
		AB1r	TCTCCCGGACTCCAGTCTG		
*A. ovis*	*msp4*	MSP4f	CCGGATCCTTAGCTGAACAGGAATCTTGC	867	[27]
		MSP4r	GGGAGCTCCTATGAATTACAGAGAATTGTTTAC		

**Table 3:** Occurrence of *Anaplasma* spp. in dairy, cashmere, and meat goats in Shaanxi province.

Factor	Production Categories		No. Tested	No. Positive (%)							
				Total	AP ^a^	AB ^b^	AO ^c^	AP + AB ^d^	AB + AO ^e^	AP + AO ^f^	AP + AB + AO ^g^
Production											
	Guanzhong dairy goat	Farm 1	26	0 (0)	0 (0)	0 (0)	0 (0)	0 (0)	0 (0)	0 (0)	0 (0)
		Farm 2	39	23 (59.0)	13 (33.3)	18 (46.2)	0 (0)	8 (20.5)	0 (0)	0 (0)	0 (0)
		Farm 3	17	1 (5.9)	1 (5.9)	1 (5.9)	0 (0)	1 (5.9)	0 (0)	0 (0)	0 (0)
		Farm 4	15	0 (0)	0 (0)	0 (0)	0 (0)	0 (0)	0 (0)	0 (0)	0 (0)
		Farm 5	27	22 (81.5)	21 (77.8)	15 (55.6)	0 (0)	14 (51.9)	0 (0)	0 (0)	0 (0)
		Farm 6	91	65 (71.4)	38 (41.8)	45 (49.5)	4 (4.4)	19 (20.9)	0 (0)	1 (1.1)	1 (1.1)
		Farm 7	12	3 (25.0)	0 (0)	3 (25.0)	0 (0)	0 (0)	0 (0)	0 (0)	0 (0)
	Saanen dairy goat	Farm 8	22	1 (4.5)	0 (0)	1 (4.5)	0 (0)	0 (0)	0 (0)	0 (0)	0 (0)
	Subtotal		249	115 (46.2)	73 (29.3)	83 (33.3)	4 (1.6)	42 (16.9)	0 (0)	1 (0.4)	1 (0.4)
	Shaanbei white cashmere goat	Farm 9	37	20 (54.1)	0 (0)	2 (5.4)	20 (54.1)	0 (0)	2 (5.4)	0 (0)	0 (0)
		Farm 10	39	31 (79.5)	8 (20.5)	23 (59.0)	18 (46.2)	3 (7.7)	6 (15.4)	1 (2.6)	4 (10.3)
		Farm 11	24	6 (25.0)	2 (8.3)	3 (12.5)	4 (16.7)	1 (4.2)	0 (0)	0 (0)	1 (4.2)
		Farm 12	39	24 (61.5)	14 (35.9)	10 (25.6)	10 (25.6)	5 (12.8)	2 (5.1)	3 (7.7)	0 (0)
	Subtotal		139	81 (58.3)	24 (17.3)	38 (27.3)	52 (37.4)	9 (6.5)	10 (7.2)	4 (2.9)	5 (3.6)
	Shaannan white goat	Farm 13	40	36 (90.0)	32 (80.0)	33 (82.5)	0 (0)	29 (72.5)	0 (0)	0 (0)	0 (0)
		Farm 14	39	34 (87.2)	33 (84.6)	10 (25.6)	0 (0)	9 (23.1)	0 (0)	0 (0)	0 (0)
		Farm 15	42	32 (76.2)	26 (61.9)	20 (47.6)	0 (0)	14 (33.3)	0 (0)	0 (0)	0 (0)
	Subtotal		121	102 (84.3)	91 (75.2)	63 (52.1)	0 (0)	52 (43.0)	0 (0)	0 (0)	0 (0)
Ages											
	<2 months		15	12 (80.0)	4 (26.7)	12 (80.0)	4 (26.7)	2 (13.3)	2 (13.3)	0 (0)	2 (13.3)
	3–6 months		99	44 (44.4)	27 (27.3)	25 (25.3)	14 (14.1)	15 (15.2)	2 (2.0)	3 (3.0)	1 (1.0)
	7–12 months		60	45 (75.0)	26 (43.3)	27 (45.0)	14 (23.3)	13 (21.7)	4 (6.7)	1 (1.7)	2 (3.3)
	>12 months		335	197 (58.8)	131 (39.1)	120 (35.8)	24 (7.2)	73 (21.8)	2 (0.6)	1 (0.3)	1 (0.3)
	Total		509	298 (58.5)	188 (36.9)	184 (36.1)	56 (11.0)	103 (20.2)	10 (2.0)	5 (1.0)	6 (1.2)

^a/b/c^*A. phagocytophilum*/*A. bovis*/*A. ovis*. ^d/e/f/g^ Co-infection of *A. phagocytophilum* and *A. bovis*/*A. bovis* and *A. ovis*/*A. phagocytophilum* and *A. ovis*/*A. phagocytophilum*, *A. bovis*, and *A. ovis*.

**Table 4:** Differences in the occurrence of *Anaplasma* in each production categories of goats in the present study, as indicated by results of chi-square analysis.

Pathogen	Meat Goat		Cashmere Goat				Dairy Goat			
	*n*	χ^2^	*n*	χ^2^	OR (95% CI)	*p*	*n*	χ^2^	OR (95% CI)	*p*
Any *Anaplasma*	102	REF	81	21.018	3.84 (2.12–6.97)	<0.001	115	48.773	6.26 (3.61–10.84)	<0.001
*A. phagocytophilum*	91	REF	24	88.038	14.54 (7.95–26.57)	<0.001	73	69.486	7.31 (4.46–11.99)	<0.001
*A. bovis*	63	REF	38	16.651	2.89 (1.72–4.84)	<0.001	83	11.962	2.17 (1.39–3.39)	0.001
*A. ovis*	0	REF	52	56.583	NA	<0.001	4	1.965	NA	0.161

OR, odds ratio; CI, confidence interval; NA, not available.

**Table 5:** Differences in the occurrence of *Anaplasma* in goats among production categories, ages, and sampling sites, as indicated by results of chi-square analysis.

Pathogen	Production Categories			Ages			Sampling Sites (All)			Sampling Sites (Positive)		
	χ^2^	*df*	*p*	χ^2^	*df*	*p*	χ^2^	*df*	*p*	χ^2^	*df*	*p*
Any *Anaplasma*	48.743	2	<0.001	17.659	3	0.001	174.903	14	<0.001	117.44	12	<0.001
*A. phagocytophilum*	105.376	2	<0.001	6.378	3	0.095	187.211	14	<0.001	96.34	9	<0.001
*A. bovis*	18.812	2	<0.001	19.641	3	<0.001	126.079	14	<0.001	98.871	12	<0.001
*A. ovis*	136.408	2	<0.001	19.113	3	<0.001	167.124	14	<0.001	48.265	4	<0.001

OR, odds ratio; CI, confidence interval; NA, not available.

**Table 6:** Association between production categories and the occurrence of *Anaplasma* in four age groups from seven sampling sites, as indicated by univariate and multivariate analyses.

Pathgen	Factor	Specimens Size of Factor (*n*)	Univariate Model			Multivariate Model		
			OR	95% CI	*p*	OR	95% CI	*p*
Any *Anaplasma* (*n* = 298)	Production categories							
	meat goat	102	0.36	0.25–0.52	<0.001	0.97	0.35–4.79	0.982
	cashmere goat	81	0.98	0.66–1.47	0.939	NA	NA	NA
	dairy goat	115	5.26	3.17–9.16	<0.001	0.79	0.48–1.27	0.532
	Ages							
	<2 months	12	2.91	0.91–12.89	0.101	0.52	0.07–4.95	0.004
	3–6 months	44	0.49	0.31–0.76	0.002	0.26	0.10–0.63	0.017
	7–12 months	45	2.32	1.29–4.42	0.007	0.27	0.09–0.79	<0.001
	>12 months	197	1.03	0.71–1.49	0.869	NA	NA	NA
	Sampling sites							
	Lantian	46	0.35	0.23–0.54	<0.001	0.98	0.36–4.82	0.982
	Linyou	68	1.49	0.95–2.36	0.086	0.97	0.32–4.73	0.981
	Mizhi	20	0.82	0.42–1.62	0.565	NA	NA	NA
	Qingjian	24	2.95	1.39–7.01	0.008	9.8	2.14–47.59	0.004
	Suide	37	0.6	0.35–1.03	0.062	2.97	0.89–10.33	0.081
	Sanyuan	1	0.98	0.82–1.60	0.971	NA	NA	NA
	Zhenba	102	5.26	3.17–9.16	<0.001	8.94	3.45–24.83	<0.001
*A. phagocytophilum* (*n* = 188)	Production categories							
	meat goat	91	9.1	5.74–14.79	<0.001	0.98	0.86–1.72	0.98
	cashmere goat	24	0.26	0.16–0.42	<0.001	0.89	0.37–2.31	0.837
	dairy goat	73	0.52	0.36–0.75	0.001	0.41	0.25–0.69	0.01
	Ages							
	<2 months	4	0.61	0.17–1.82	0.407	NA	NA	NA
	3–6 months	27	0.58	0.35–0.93	0.028	0.89	0.29–2.68	0.836
	7–12 months	26	1.35	0.78–2.33	0.276	NA	NA	NA
	>12 months	131	1.32	0.9–1.94	0.16	3.23	1.32–8.04	<0.001
	Sampling sites							
	Lantian	35	0.64	0.4–0.98	0.045	1.17	0.67–2.05	0.575
	Linyou	38	0.99	0.92–1.85	0.992	NA	NA	NA
	Mizhi	0	0.97	0.85–1.72	0.979	NA	NA	NA
	Qingjian	14	0.42	0.17–0.88	0.031	0.96	0.67–1.82	0.979
	Suide	10	0.54	0.29–0.97	0.045	0.96	0.57–1.79	0.978
	Sanyuan	0	0.97	0.77–1.59	0.973	NA	NA	NA
	Zhenba	91	9.1	5.74–14.79	<0.001	12.84	6.53–26.8	<0.001
*A. bovis* (*n* = 184)	Production categories							
	meat goat	63	2.4	1.58–3.64	<0.001	1.97	0.96–4.82	0.089
	cashmere goat	38	0.58	0.37–0.88	0.012	1.28	0.47–2.77	0.423
	dairy goat	83	0.79	0.55–1.13	0.196	0.45	0.21–0.86	0.314
	Ages							
	<2 months	12	7.49	2.34–33.19	0.002	5.06	0.88–42.36	<0.001
	3–6 months	25	0.53	0.32–0.86	0.013	0.73	0.32–1.56	0.645
	7–12 months	27	1.52	0.88–2.62	0.131	0.66	0.3–1.47	0.578
	>12 months	120	0.96	0.66–1.41	0.831	NA	NA	NA
	Sampling sites							
	Lantian	34	0.66	0.42–1.03	0.07	1.03	0.51–2.41	0.982
	Linyou	48	1.73	1.12–2.69	0.014	1.05	0.63–2.67	0.982
	Mizhi	2	0.09	0.01–0.3	0.001	1.05	0.66–2.83	0.985
	Qingjian	10	2.76	1.43–5.46	0.003	0.97	0.65–1.43	0.981
	Suide	26	0.42	0.21–0.77	0.008	0.98	0.77–1.69	0.983
	Sanyuan	1	0.97	0.83–1.69	0.973	NA	NA	NA
	Zhenba	63	2.4	1.58–3.64	<0.001	0.97	0.89–1.87	0.981
*A. ovis* (*n* = 56)	Production categories							
	meat goat	0	0.98	0.86–1.65	0.985	NA	NA	NA
	cashmere goat	52	9.86	3.69–24.18	<0.001	0.98	0.37–4.01	0.937
	dairy goat	4	0.07	0.02–0.16	<0.001	1.02	0.57–2.36	0.981
	Ages							
	<2 months	4	3.09	0.83–9.41	0.061	1.03	0.49–2.45	0.986
	3–6 months	14	1.44	0.73–2.7	0.268	NA	NA	NA
	7–12 months	14	2.95	1.46–5.7	0.002	1.04	0.63–2.71	0.986
	>12 months	24	0.34	0.19–0.6	<0.001	0.96	0.32–3.97	0.932
	Sampling sites							
	Lantian	0	0.98	0.89–1.77	0.986	NA	NA	NA
	Linyou	4	0.28	0.08–0.69	0.015	1.03	0.41–2.09	0.991
	Mizhi	20	14.25	6.89–29.94	<0.001	0.97	0.41–4.07	0.992
	Qingjian	10	9.74	4.76–19.91	<0.001	0.98	0.37–3.89	0.994
	Suide	22	2.75	1.36–5.29	0.003	0.98	0.42–3.88	0.995
	Sanyuan	0	0.96	0.66–1.71	0.984	NA	NA	NA
	Zhenba	0	0.96	0.71–1.69	0.985	NA	NA	NA

OR, odds ratio; CI, confidence interval; NA, not available.

## Data Availability

Data is contained within the article.
